# 3,4-Dibromo-7-Azaindole Modulates Arabidopsis Circadian Clock by Inhibiting Casein Kinase 1 Activity

**DOI:** 10.1093/pcp/pcz183

**Published:** 2019-09-14

**Authors:** Azusa Ono, Ayato Sato, Kazuhiro J Fujimoto, Hiromi Matsuo, Takeshi Yanai, Toshinori Kinoshita, Norihito Nakamichi

**Affiliations:** 1 Division of Biological Science, Graduate School of Science, Nagoya University, Furocho, Chikusa, Nagoya, Japan; 2 Institute of Transformative Bio-Molecules (WPI-ITbM), Nagoya University, Furocho, Chikusa, Nagoya, Japan; 3 Department of Chemistry, Graduate School of Science, Nagoya University, Furocho, Chikusa, Nagoya, Japan

**Keywords:** *Arabidopsis thaliana* (Arabidopsis), Casein Kinase 1, Chemical Screening, Circadian clock

## Abstract

The circadian clock is a timekeeping system for regulation of numerous biological daily rhythms. One characteristic of the circadian clock is that period length remains relatively constant in spite of environmental fluctuations, such as temperature change. Here, using the curated collection of in-house small molecule chemical library (ITbM chemical library), we show that small molecule 3,4-dibromo-7-azaindole (B-AZ) lengthened the circadian period of *Arabidopsis thaliana* (Arabidopsis). B-AZ has not previously been reported to have any biological and biochemical activities. Target identification can elucidate the mode of action of small molecules, but we were unable to make a molecular probe of B-AZ for target identification. Instead, we performed other analysis, gene expression profiling that potentially reveals mode of action of molecules. Short-term treatment of B-AZ decreased the expression of four dawn- and morning-phased clock-associated genes, *CIRCADIAN CLOCK-ASSOCIATED 1* (*CCA1*), *LATE ELONGATED HYPOCOTYL* (*LHY*), *PSEUDO-RESPONSE REGULATOR 9* (*PRR9*) and *PRR7*. Consistently, amounts of PRR5 and TIMING OF CAB EXPRESSION 1 (TOC1) proteins, transcriptional repressors of *CCA1*, *LHY*, *PRR9* and *PRR7* were increased upon B-AZ treatment. B-AZ inhibited Casein Kinase 1 family (CK1) that phosphorylates PRR5 and TOC1 for targeted degradation. A docking study and molecular dynamics simulation suggested that B-AZ interacts with the ATP-binding pocket of human CK1 delta, whose amino acid sequences are highly similar to those of Arabidopsis CK1. B-AZ-induced period-lengthening effect was attenuated in *prr5 toc1* mutants. Collectively, this study provides a novel and simple structure CK1 inhibitor that modulates circadian clock via accumulation of PRR5 and TOC1.

## Introduction

Circadian clocks are biological timekeeping systems that allow organisms to coordinate their activities with daily fluctuations such as light–dark and warm–cold cycles that originate from earth’s rotation. Although fundamental properties of circadian clocks (a period of about 24 h under constant conditions can be entrained by the environmental time cues, and period length is robust against environmental fluctuations) are well conserved among bacteria, fungi, plants and animals, clock components are different among phylogenetic lineages (Nohales and Kay 2016). Cyanobacteria employ the KaiC activity cycle as the clock; time information governed by KaiC controls the activity of transcriptional factors, leading to genome-wide rhythmic gene expression (Nakajima et�al. 2005, Takai et�al. 2006, Markson et�al. 2013). In eukaryotes, transcription translation feedback loop is essential for clock function. The mechanism for plant circadian clocks was proposed to be a transcription translation feedback loop with a repressilator-like structure in which three classes of transcriptional factors repress transcription of genes expressed during earlier phases (Nakamichi 2011, Pokhilko et�al. 2012), but recent progresses have demonstrated that the transcription translation feedback loop is highly wired network (Millar 2016, Nohales and Kay 2016). The network components are modulated at least at the level of transcription and post-translational modifications, by light and temperature changes as environmental time cues (Inoue et�al. 2017). The transcription translation feedback loop controls the circadian rhythms of many physiological processes through directly regulating the expression of key genes in output pathways (Huang et�al. 2012, Nakamichi et�al. 2012, Nagel et�al. 2015, Kamioka et�al. 2016, Liu et�al. 2016, Ezer et�al. 2017, Adams et�al. 2018). Network architectures are partly conserved among flowering plants (Toda et�al. 2019), but divergence is important for adaptation (Itoh et�al. 2019).

In the transcription translation feedback loop of *Arabidopsis thaliana* (Arabidopsis), three classes of transcriptional repressors together form a repressilator-like structure. CIRCADIAN CLOCK-ASSOCIATED 1 (CCA1) and LATE ELONGATED HYPOCOTYL (LHY) are expressed at dawn and encode single Myb-type transcription factors that repress day-time expressed *PSEUDO-RESPONSE REGULATOR*s (*PRR*s) and evening to nighttime-expressed *LUXARRHYTHMO* (*LUX*), *EARLY FLOWERING 3* (*ELF3*) and *ELF4* (Alabadi et�al. 2001, Nagel et�al. 2015, Kamioka et�al. 2016, Adams et�al. 2018). The PRR family [PRR9, PRR7, PRR5 and TIMING OF CAB EXPRESSION 1 (TOC1), called as PRR1] encode transcriptional repressors that directly repress *CCA1*, *LHY* and *PRR*s expressed during earlier phases (Nakamichi et�al. 2010, Gendron et�al. 2012, Nakamichi et�al. 2012, Liu et�al. 2013, Liu et�al. 2016). LUX, ELF3 and ELF4 proteins form the Evening Complex that represses *LUX*, *PRR9* and *PRR7* expression (Dixon et�al. 2011, Helfer et�al. 2011, Nusinow et�al. 2011, Ezer et�al. 2017). In addition to the repressilator-like loop, REVIREE8 (RVE8) and NIGHT LIGHT-INDUCIBLE AND CLOCK-REGULATED GENE 1 (LNK1) activate *PRR5* and *TOC1* expression (Rawat et�al. 2011, Rugnone et�al. 2013, Ma et�al. 2018, Shalit-Kaneh et�al. 2018). PRR9, PRR7, PRR5 and TOC1 in turn repress *RVE*s and *LNK*s genes (Nakamichi et�al. 2012, Rugnone et�al. 2013). TEOSINTE BRANCHED 1-CYCLOIDEA-PCF20 (TCP20) and TCP22, and LIGHT-REGULATED WD 1 (LWD1) form complexes and activate the expression of *CCA1* (Wu et�al. 2016). *CCA1-HIKING EXPEDITION* (*CHE*) encodes TCP transcription factor, and represses *CCA1* (Pruneda-Paz et�al. 2009). CCA1 and LHY repress *CHE* expression. In addition to transcription translation feedback loop, post-translational regulation is involved in the clock. Phosphorylation of CCA1 and LHY by Casein Kinase 2 (CK2) is crucial for DNA-binding activities of CCA1 and LHY (Sugano et�al. 1999, Daniel et�al. 2004). ZEITLUPE (ZTL), a component of the ubiquitin E3 ligase Skp-Cullin-F-box complex degrades PRR5 and TOC1 preferentially in the dark (Mas et�al. 2003, Kiba et�al. 2007). The phosphorylated forms of PRR5 and TOC1 are bound by ZTL for targeted degradation (Fujiwara et�al. 2008). Phosphorylation of PRR3 and TOC1 triggers their interaction and subsequently inhibits recognizing by ZTL. Thus, there are multiple phosphorylations sites on TOC1 that regulate degradation and stabilization (Fujiwara et�al. 2008).

Many plant lineages have undergone whole genome duplications during evolution, by which plants enrich functionally redundant genes in their genomes (The Arabidopsis Genome Initiative 2000). This may make further discoveries of clock-associated genes technically difficult by forward genetic approaches. To identify such potentially redundant genes that are involved in the clock, screening of small molecules and revealing mode of action of these molecules have emerged as the preferred methodology. Natural compounds that affect actin-associated processes also influence Arabidopsis clock period (Toth et�al. 2012). PHA767491, originally found as mammal cell division cycle 7 (CDC7) and cyclin-dependent kinase 9 (CDK9) inhibitor can lengthen the period of Arabidopsis clock (Uehara et�al. 2019). In Arabidopsis, PHA767491 inhibits Casein Kinase 1-like (CKL) family that consists of 13 members. Given that PHA767491 treatment caused the accumulation of PRR5 and TOC1 in vivo and inhibited CKL4-dependent phosphorylation of PRR5 and TOC1 in vitro, it was suggested that highly redundant CKLs phosphorylate PRR5 and TOC1 for degradation (Uehara et�al. 2019).

In this study, we performed a chemical screening using the ITbM chemical library (Ziadi et�al. 2017, Toh et�al. 2018) and found a compound with a relatively simple structure, 3,4-dibromo-7-azaindole (B-AZ). Our primary structure–activity relationship study of B-AZ suggested that target identification using a molecular probe was not possible; however, gene expression profiling after short-term treatment with B-AZ suggested that B-AZ immediately controls the expression of *CCA1*, *LHY*, *PRR9* and *PRR7*. B-AZ treatment resulted in accumulation of PRR5 and TOC1 proteins that act as repressors for *CCA1*, *LHY*, *PRR9* and *PRR7*. B-AZ inhibited CKL kinase that controls PRR5 and TOC1 protein amounts, emphasizing that inhibition of CKL is one of the pharmacologically controllable steps in the clock.

## Results and Discussion

### Screening of small molecules that can change the circadian period

We searched for small molecules that could regulate circadian clock, from the ITbM chemical library, our unique chemical library that was enriched with plant hormone mimic molecules for use in plant-based phenotypic screening (Ziadi et�al. 2017, Toh et�al. 2018). We monitored the circadian rhythms of transgenic plants harboring a clock reporter [*CCA1:Luciferase (LUC)*, exhibiting circadian bioluminescence rhythm that peaks in the morning] upon treatment with small molecules from the library. Although most small molecules did not influence the circadian period, B-AZ lengthened the circadian period of *CCA1:LUC* (Fig.�1A). Note that another parameter of circadian rhythm, the amplitude, was highly variable among the samples in our screening system, so that screening molecules for changing amplitudes seemed technically difficult (Fig.�1B). Seedling size was not likely the reason for the differing amplitudes because we carefully selected seedlings of similar size at 4 d after germination. Rather, we hypothesize that other factors, such as fluctuation of temperature during screening, may have affected the amplitude. This hypothesis is consistent with general properties of circadian rhythms; period length is robust against environmental fluctuation, but amplitude is variable (Murayama et�al. 2017). We have found some other small molecules from the ITbM chemical library that changed the circadian period and will report these molecules in future studies.

**Fig. 1:**
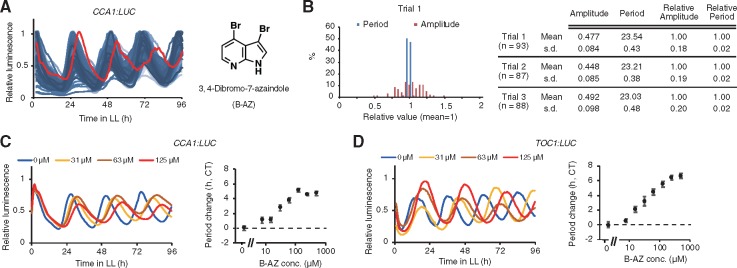
B-AZ lengthens circadian period of Arabidopsis. (A) The screening result showing that B-AZ has the potential to lengthen the circadian period of Arabidopsis seedling (left). Traces of bioluminescence of *CCA1:LUC* seedlings treated with random small molecules showed similar period length (blue traces), except for a sample treated with 50 �M B-AZ (red trace). Structure of B-AZ (right). (B) Amplitude and period in three independent trials (*n *= 87–93) under constant light conditions were determined by a CL96-attached software. (C) Averaged traces of relative luminescence of *CCA1:LUC* (left) and period length (mean � SEM, *n *= 8, right). (D) Averaged traces of relative luminescence of *TOC1:LUC* (left) and period length (mean � SEM, *n *= 5 or 6, right).

To validate the period-lengthening activity of B-AZ, we further analyzed the *CCA1:LUC* luminescence upon the continuous treatment of different concentration of B-AZ (Fig.�1C). B-AZ lengthened the circadian period of *CCA1:LUC* reporter in a dose-dependent manner. B-AZ lengthened the period of *CCA1:LUC* about 5 h at 125 �M. B-AZ also lengthened the circadian period of other clock reporter line, *TOC1:LUC* (exhibiting circadian luminescence rhythm that peaks in the evening), validating that B-AZ lengthens the period of the Arabidopsis circadian clock (Fig.�1D). We also noticed that the period length in samples treated with 250 and 500 �M B-AZ were similar (about 6 h longer than the control samples), suggesting that period lengthening seemed to be saturated at concentration of B-AZ over 250 �M. By validation using two clock reporters, we confirmed that B-AZ lengthens circadian clock of Arabidopsis. Putative biological activities of B-AZ besides period-lengthening activity are intriguing and will be examined in a future study.

Although the structure of B-AZ is partly similar to plant hormone cytokinin, we hypothesized that B-AZ lengthens clock period through cytokinin-independent manner. This hypothesis is probably correct since the application of cytokinin does not result in lengthening or shortening the circadian period (Hanano et�al. 2006). Secondly, the side-chain structures of cytokinin are essential for their biological activity (Sakakibara 2006), but B-AZ lacks such side chain (Fig.�1A).

To reveal the mode of action of B-AZ, screening for proteins capable of being bound by a small molecule (Target Identification) is often regarded as a first and crucial step (Hirota et�al. 2010, Dejonghe and Russinova 2017). To this end, generating the molecule that is covalently attached to bead is required. To understand which position in the molecule should be the site for conjugating the molecular linker, we again checked first screening result since molecules whose structures are partially similar to B-AZ were in the ITbM chemical library. We found no period-lengthening activity of these molecules (Fig.�2A). B-AZ has two bromine atoms at 3 and 4 positions. To test the importance of these bromines, and possibly install linker molecule at these positions, molecules lacking the bromines (7-azaindole, 3-bromo-7-azaindole and 4-bromo-7-azaindole) were treated with seedlings and the circadian rhythms were analyzed (Fig.�2B). There was no obvious period-lengthening effect by 7-azaindole or 4-bromo-7-azaindole. A weak period-lengthening effect was observed by 3-bromo-7-azaindole and may suggest importance of the bromine in the B-AZ. Collectively, these results indicated that generating molecular probe for target identification of B-AZ is difficult.

**Fig. 2:**
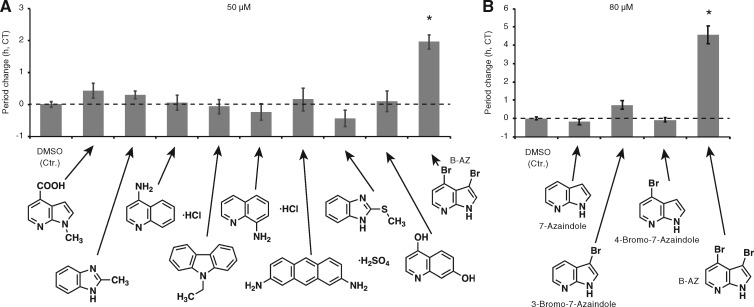
Period length of seedlings treated with B-AZ analogs. The circadian period of *CCA1:LUC* treated with small molecules similar to B-AZ (50 �M) (A) or (80 �M) (B) (mean � SEM, *n *> 6). Asterisks indicate a significant period change compared to the DMSO control (Student’s *t*-test *P* < 0.01). Similar results were obtained in other trials.

### Short-term B-AZ treatment downregulates CCA1, LHY, PRR9 and PRR7

To understand the mode of action of B-AZ for clock period lengthening in other ways, we focused on gene expression profiling after a short-term B-AZ treatment. Even if the expression changes were not drastic, this approach would reveal an immediate effect on gene expression resulting from the B-AZ treatment, and would allow us to speculate the state of clock-associated transcription factors that potentially regulate these immediate altered genes.

Arabidopsis seedlings were grown under 12 h light/12 h dark conditions (LD) for 4 d, and transferred into constant light, and treated with B-AZ at eight time points (25, 28, 31, 34, 37, 40, 43 and 46 h after being moved to the constant light conditions, Fig.�3A). Plants were sampled 3 h after treatment. Reverse transcription quantitative PCR (RT-qPCR) analysis indicated that *LUX* expression was not changed by B-AZ treatment. With the exception of time-point 28 h, *TOC1* expression was also not changed by B-AZ. B-AZ treatment decreased *ELF4* expression at around subjective noon and early night. B-AZ decreased *PRR5* from subjective night to early morning. B-AZ significantly decreased *PRR7* and *PRR9* expression in subjective night and highly suppressed their peaks. B-AZ decreased *CCA1* and *LHY* in subjective early night, before the peak time for expression of these genes.

**Fig. 3:**
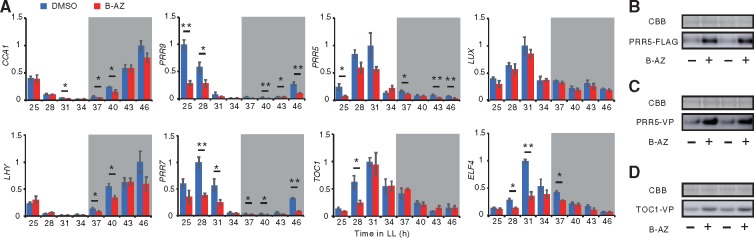
Clock-associated genes expression upon B-AZ treatment. (A) Expression of clock-associated genes in seedlings treated with B-AZ for 3 h (mean � SEM, *n *= 3). Asterisks and double asterisks indicate significant differences between solvent DMSO and B-AZ-treated samples (Student’s *t*-test *P* < 0.05 and 0.01, respectively). Time indicates hours after transfer to continuous light. White and gray areas were subject day and night, respectively. (B–D**)** PRR fusion proteins in seedlings treated with B-AZ were analyzed by Western blotting (lower). Coomassie brilliant blue (CBB) staining indicates similar amounts of total proteins were analyzed (upper). Similar results were obtained in other experiments for (A–D).

Collectively, B-AZ did not affect all of clock-associated genes expression. Rather, B-AZ had a lesser effect on the evening-phased genes such as *TOC1* and *LUX*. B-AZ reduced *CCA1, LHY, PRR9* and *PRR7* at time when their expression would normally start to increases and also decreased *PRR9* and *PRR7* in subjective morning when their expression should peak.

### B-AZ increases the amounts of PRR5 and TOC1 proteins

Given that PRR proteins are transcriptional repressors for *CCA1*, *LHY*, *PRR9* and *PRR7*, we hypothesized that B-AZ affects PRR proteins that eventually decrease the expression of *CCA1*, *LHY*, *PRR9* and *PRR7* genes. This hypothesis is supported by evidence showing that PHA767491, another small molecule that downregulates *CCA1*, *LHY*, *PRR9* and *PRR7*, causes increasing of amounts of two PRR proteins, PRR5 and TOC1 (Uehara et�al. 2019).

To test whether B-AZ affects the amounts of PRR proteins without regulating transcription, transgenic plants overexpressing *PRR* under the control of the CaMV 35 promoter were used (Nakamichi et�al. 2012). *35Spro:PRR5-FLAG* seedlings grown under LD conditions were treated with B-AZ and further kept under constant dark. We found that B-AZ treatment caused accumulation of PRR5-FLAG under dark conditions (Fig.�3B). The result suggested that degradation of PRR5 is attenuated by B-AZ; however, it is possible that enhancement of the transcriptional repressor function of PRR5 for the target genes that perturb clock may influence B-AZ’s activity for regulating the amount of PRR5 protein. To examine this possibility, we analyzed PRR5-VP protein in *35Spro:PRR5-VP* seedlings that have opposite phenotypes to that of *35Spro:PRR5-FLAG* (Nakamichi et�al. 2012). PRR5-VP amounts were also increased by B-AZ treatment under constant dark conditions (Fig.�3C), suggesting that PRR5-dependent transcriptional regulation of target genes less affects B-AZ’s activity to regulate PRR5 amount. Next, we analyzed the amount of TOC1-VP protein in *35Spro:TOC1-VP* seedlings (Nakamichi et�al. 2016). B-AZ caused increasing of TOC1-VP amount (Fig.�3D). These results indicate that B-AZ controls the amount of PRR5 and TOC1 proteins, as did PHA767491.

### B-AZ inhibits CKL4, a kinase that phosphorylates PRR5 and TOC1

Phosphorylated PRR5 protein is preferentially recognized by ZTL for degradation (Fujiwara et�al. 2008). Recently, we reported that Casein Kinase 1 (CK1) family protein (CKL4) phosphorylates PRR5 and TOC1 in vitro, and inhibition of CK1 activity by PHA767491 causes increasing amount of PRR5 and TOC1 proteins in vivo (Uehara et�al. 2019). We hypothesized that B-AZ also inhibits CKL activity, thereby eventually leading to an increase in PRR5 and TOC1 amount in vivo, although the structure of B-AZ is not similar to that of PHA767491.

The CKL4 kinase activity for model substrate casein was analyzed in vitro, since CKL4 kinase activity was strongest among the purified CKL proteins (Uehara et�al. 2019). B-AZ inhibited CKL4 kinase activity with an IC_50_ around 40 �M, far lower than the concentration required for period lengthening in vivo (Fig.�4A). B-AZ also inhibited CKL1 kinase activity (Fig.�4A). B-AZ analogs lacking at least one bromine (7-azaindole, 3-bromo-7-azaindole or 4-bromo-7-azaindole) had very weak CKL4 inhibitory activities, suggesting that two bromines are essential for CKL4 inhibitory activity (Fig.�4B). The correlation of structure–activity relationship studies with the in vitro CKL4 kinase assay and in vivo period-lengthening effect support the idea that B-AZ lengthens the period through CKL inhibition.

**Fig. 4:**
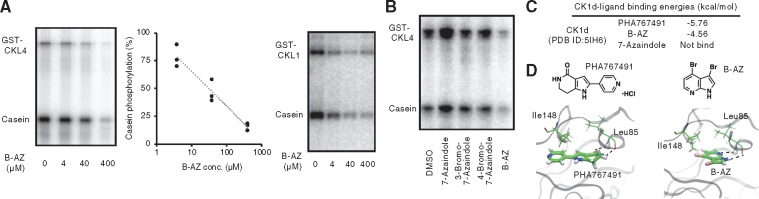
Inhibition of CKL4 activity by B-AZ in vitro. (A**)** Autoradiography of in vitro kinase activity of CKL4 with B-AZ (left). The graph for the IC_50_ of B-AZ on CKL4, determined by three separate experiments (middle). Autoradiography of in vitro kinase activity of CKL1 with B-AZ (right). Inhibition of CKL1 kinase activity by B-AZ was observed in other experiments. (B) Effect of B-AZ analogs (40 �M) of CKL4 kinase activity in vitro. (C) Binding energy between human CK1 delta (Protein Data Bank ID, PDB ID: 5IH6) and small molecules. (D) Binding structure of PHA767491 (left) or B-AZ (right) in human CK1 delta ATP-binding pocket. Leu85 and Ile148 of CK1 delta are shown. Blue in ligands means nitrogen atom. Red in PHA767491 and B-AZ means oxygen and bromine atoms, respectively. Dashed line indicates hydrogen bond between Leu85 and the ligand.

To further understand how B-AZ inhibits CKL, molecular docking and molecular dynamics (MD) simulations were performed to predict B-AZ binding site in human CK1 delta (PDB ID: 5IH6, Ursu et al. 2016), whose amino acid sequences are highly similar to those of Arabidopsis CKLs (Supplementary Fig. S1), since crystal structures of Arabidopsis CKLs have not been reported. To validate our strategy to use human CK1 delta, we first analyzed whether PHA767491 binds to CK1 delta. The in vitro inhibitory activity of PHA767491 on CK1 delta and the PHA767491-dependent period lengthening of mammal circadian clock were shown, but the actual inhibitory mechanism of PHA767491 on CK1 delta was unclear (Uehara et�al. 2019). The computational results successfully provided the binding conformation of PHA767491 in the ATP-binding pocket of CK1 delta (Fig.�4C, D). The binding energy between PHA767491 and CK1 delta was calculated to be −5.76 kcal/mol (Fig.�4C). The MD simulation also suggested that the binding structure of PHA767491 in the CK1 delta ATP-binding pocket is stable (Fig.�4D). PHA767491 spatially associates with two amino acid residues, Leu85 and Ile148 in the ATP-binding pocket of CK1 delta. PHA767491 binds to Leu85 via a hydrogen bond. Then, the same analysis was applied to B-AZ, resulting in a binding conformation in the ATP-binding pocket (Fig.�4C) and the binding energy of −4.56 kcal/mol. This value showed higher binding energy than that for PHA767491, suggesting that binding between PHA767491 and CK1 delta is stronger than that between B-AZ and CK1 delta. The in silico results may meet with IC_50_ value of PHA767491 on CKL4 (∼5 �M) (Uehara et�al. 2019), far lower than that of B-AZ (Fig.�4A). B-AZ also has hydrogen bonds to Leu85 in the ATP-binding pocket of CK1 delta, although chemical structures of B-AZ and PHA767491 are not similar. We also applied the same computational analysis to the 7-azaindole, but failed to produce a binding conformation in the ATP-binding pocket. Collectively, it was suggested that B-AZ well binds to the ATP-binding site of CK1 delta. Binding energies between human CK1 delta were well correlated to inhibitory activities for Arabidopsis CKL4 in vitro (Fig.�4A, B) (Uehara et�al. 2019). Leu85 and Ile148 are conserved among CK1 delta and CKLs (Supplementary Fig. S1), implying that B-AZ binds to the ATP-binding site of Arabidopsis CKLs; however, future co-crystallization and MD simulation studies using B-AZ and CKL are required to confirm the mechanism of B-AZ inhibition of CKL kinase activity.

### prr5 toc1 is hyposensitive to B-AZ treatment

Our results showing that PRR5 and TOC1 proteins were accumulated by B-AZ treatment suggested that PRR5 and TOC1 are crucial factor in mode of action of B-AZ. If so, Arabidopsis lacking *PRR5* and *TOC1* should be hyposensitive to B-AZ. To examine this possibility, we treated *prr5 toc1* mutants with B-AZ and analyzed the circadian rhythm (Fig.�5A). As mentioned, 40–100 �M of B-AZ lengthened period of wild type for 4 h, and about 200 �M lengthened it for 6 h. In *prr5 toc1*, 40–100 �M lengthened below 1 h, and 200 �M lengthened 2 h, showing the hypo-sensitivity of B-AZ in period lengthening in *prr5 toc1*. There was no statistical significance of B-AZ-dependent period-lengthening sensitivities of *prr5* and *toc1* single mutants treated with 80 �M B-AZ (Fig.�5B). Genetic redundancy may mask the sensitivity of these single mutants (Uehara et�al. 2019). In short, PRR5 and TOC1 are major mediators in the mode of action of B-AZ, but other mechanisms are not excluded since B-AZ-sensitivity was not completely diminished in the *prr5 toc1* mutants. Other B-AZ target proteins than CK1 family and other CK1 substrates besides PRR5 and TOC1 may contribute to the mode of action of B-AZ.

**Fig. 5:**
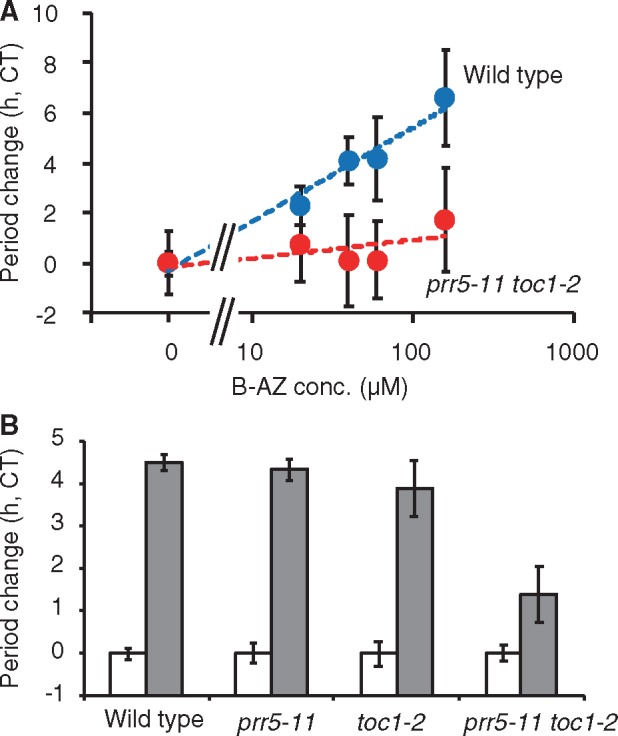
Period-lengthening effect of B-AZ in *prr5-11 toc1-2*. (A) Different concentrations of B-AZ were treated to the *prr5-11 toc1-2* mutant seedlings (mean � SEM, *n *> 8). (B) Period lengths of *prr5-11*, *toc1-2*, *prr5-11 toc1-2* treated with 80 �M B-AZ (mean � SEM, *n *> 6).

## Conclusion

In this study, we found that a structurally simple molecule, named B-AZ, has an activity to lengthen the circadian period in Arabidopsis. Although primary structure–activity relationship study suggested that generating molecular probe for direct target identification of B-AZ was difficult, gene expression profiling helped to reveal the mode of action of B-AZ.

B-AZ lengthens the period of the clock by inhibiting CK1 protein that regulates PRR5 and TOC1 amounts. This mode of action is the same as that described for PHA767491, recently identified as Arabidopsis CK1 inhibitor (Uehara et�al. 2019). In addition, the docking study and MD simulation demonstrate that B-AZ interacts with the ATP-binding pocket of CK1. Thus, these two studies emphasize that inhibition of CK1 is one of the pharmacologically controllable steps for period tuning in Arabidopsis.

Utilization of small molecules for understanding biological systems are emerging and expanding (Dejonghe and Russinova 2017, Kinoshita et�al. 2018). For instance, synthetic small molecules that modulate auxin metabolism, transport and signaling have helped us to understand auxin biology (Fukui and Hayashi 2018, Uchida et�al. 2018, Yamada et�al. 2018). Also, the molecular mechanisms of brassinosteroid biosynthesis and signaling have been revealed by extensive studies using small molecules (Asami and Yoshida 1999, De Rybel et�al. 2009, Dejonghe et�al. 2014, Yamagami et�al. 2017). Not only in hormone biology but also in a wide range of plant physiological processes, the finding molecules that perturb biological processes were used as the first step to elucidate the molecular mechanism underlying the physiology of interest (Matsubayashi et�al. 1997, Park et�al. 2009, Noutoshi et�al. 2012, Nakano et�al. 2018). Although this study failed to generate molecular probe (Fig.�2), generating molecular probes help us to find exact targets of biologically active molecules (Matsubayashi et�al. 2002, Kinoshita et�al. 2005, Sharma and Russinova 2018, Tsuchiya 2018, Uehara et�al. 2019). Full understanding of the mode of action of biologically active molecules eventually expands our knowledge of plant cell signaling at the molecular level.

As the clock regulates a wide range of physiological processes such as photosynthesis, cell elongation and flowering time regulation, further discovery and development of small molecules controlling clock may provide plant growth regulators (Uehara et�al. 2019).

## Materials and Methods

### Plant materials and growth conditions

Derivatives of *A. thaliana* accession Columbia (Col-0) were used in this study. *CCA1:LUC* (Nakamichi et�al. 2005), *TOC1:LUC* (Uehara et�al. 2019), *35Spro:PRR5-FALG*, *35Spro:PRR5-VP* (Nakamichi et�al. 2012), *35Spro:TOC1-VP* (Nakamichi et�al. 2016), *prr5-11 CCA1:LUC* (Nakamichi et�al. 2005), *toc1-2 CCA1:LUC* (Ito et�al. 2009) and *prr5-11 toc1-2 CCA1:LUC* (Uehara et�al. 2019) were described previously. Plants were grown on Murashige Skoog (MS) medium (pH 5.7) (Murashige and Skoog 1962) with 0.25% sucrose and 0.3% gellan gum. Plates were stored at 4�C on dark for 2 d, and moved to LD or constant light conditions (LL). Light intensity was ∼70 �mol s^−^^1^m^−^^2^.

### Chemical screening for molecules that affect period length

Screening of small molecules changing circadian period was performed as previously described (Uehara et�al. 2019), using an ITbM chemical library (Ziadi et�al. 2017, Toh et�al. 2018), in which all molecules were dissolved in dimethyl sulfoxide (DMSO, Molecular biology grade, Nacalai, Japan), *CCA1:LUC* transgenic seedlings and an automated luminescence monitoring system (CL96, Churitsu). Molecules dissolved in DMSO at 1 mM were diluted with half strength of MS media to 50 �M, and dropped on 4-day-old seedling grown under LD. Period length of *CCA1:LUC* was determined by CL96-attached software (Churitsu, Japan), as described previously (Kamioka et�al. 2016). After the first screening, the hit molecule (B-AZ) was tested in different concentrations to period-lengthening effects of *CCA1:LUC* and *TOC1:LUC*. Other hit molecules discovered in this project will be described in future. B-AZ was also purchased from SINOVA Inc., Maryland. 7-Azaindole, 3-bromo-7-azaindole and 4-bromo-7-azaindole were purchased from Sigma-Aldrich, Tokyo Kasei and Fujifilm-Wako, respectively. The sensitivity of B-AZ in *prr5-11 CCA1:LUC*, *toc1-2 CCA1:LUC* and *prr5-11 toc1-2 CCA1:LUC* was performed by the same method. Period lengths were normalized to the period length of each genotype treated with DMSO solvent control, since period length of the mutants was shorter than that of the wild type.

### Effect of B-AZ on gene expression

Seedlings were germinated and grown on MS for 4 d under LD conditions, and transferred to LL. Then, seedlings were transferred into a conical tube and treated with 50 �M of B-AZ for 3 h. RNA isolation, RT-qPCR were done by methods reported previously (Nakamichi et�al. 2012). We used three biological replicates for each sample.

### Effect of B-AZ on PRR protein

*35Spro:PRR5-FLAG* and *35Spro:TOC1-VP* seedlings were germinated and grown on MS for 4 d under LD. *35Spro:TOC1-VP* was grown under constant light conditions. Then, seedlings with MS medium were transferred into a well of 96-well plate by a dropper, and treated with 500 �M of B-AZ and kept under constant dark conditions for 16–28 h. Thirty seedlings were gathered as one biological replicate, and frozen by liquid nitrogen. Isolation of total protein from froze samples was performed as described previously (Uehara et�al. 2019). Detection of FLAG or VP fusion proteins was done as previously described (Nakamichi et�al. 2012, Nakamichi et�al. 2016).

### In vitro CKL kinase assay

Recombinant glutathione S-transferase (GST)-CKL4, GST-CKL1, casein, B-AZ and [γ-^32^P] ATP (NN-NEG502A, PerkinElmer) were used for in vitro kinase assay, as described previously (Uehara et�al. 2019). The IC_50_ (the half-maximal inhibitory concentration) of CKL4 kinase activity from the means of three separate experiments was calculated, as described previously (Uehara et�al. 2019).

### In silico study

Molecular docking simulations were performed with F*l*ABCps (Uehara et�al. 2015), where the AutoDock force field (Huey et�al. 2007) was employed for evaluating the energy score. For accurate estimations of the binding energy, 100 ns MD simulations were performed on a periodic boundary box (80 � 82 � 88 �^3^) composed of human CK1 delta (PDB ID: 5IH6, Ursu et al. 2016), ligands and water molecules, using a time step of 2 fs under NPT conditions at 300 K and 1 atm. The computational settings of molecular docking and MD simulations were the same as in a previous study (Fujimoto et�al. 2018). All MD simulations were performed with the AMBER16 program package (Case et�al. 2016). Alignment of CKLs and human CK1 delta kinase domains was performed by ClustraIW tool in DNA Data Bank of Japan (DDBJ, https://www.ddbj.nig.ac.jp/index-e.html), with default setting.

## Funding

This study was partly supported by Japan Society for the Promotion of Science Grants-in-Aid for Scientific Research 17K19229, 18H02136 (to N.N.), Grant-in-Aid for Scientific Research on Innovative Areas 15H05956 (to T.K. and N.N.) and Toyota Riken Scholar (to N.N.). ITbM is supported by the World Premier International Research Center Initiative (WPI), Japan.

## Supplementary Material

pcz183_Supplementary_Data
