# Supplementary material for: 3,4-Dibromo-7-Azaindole Modulates Arabidopsis Circadian Clock by Inhibiting Casein Kinase 1 Activity
Source: Plant Cell Physiol. 2019 Sep 14;60(11):2360–8. doi: 10.1093/pcp/pcz183 (PMC6839374; doi:10.1093/pcp/pcz183)
Supplement: pcz183_Supplementary_Data [file pcz183_supplementary_data.pdf]

```
CKL1     FRLGRKIGSGSFGEIYLGTNIHTNEELAIKLENVKTKHPQLLYESKLYRILQGGTGVPNV 60
CKL12    YRLGRKIGSGSFGEIYLGTHIQTNEEVAIKLENVKTKHPQLLYESKLYRILQGGTGVPNI 60
CKL2     FRLGRKIGGGSFGEIYLGTNIQTNEEVAIKLENVKTKHPQLLYESKLYKVLQGGTGVPNV 60
CKL5     FRLGRKIGSGSFGEIYLGTDVQTNEEVAIKLESVKTAHPQLSYESRIYRVLQGGTGIPNM 60
CK1d     YRLGRKIGSGSFGDIYLGTDIAAGEEVAIKLECVKTKHPQLHIESKIYKMMQGGVGIPTI 60
CKL8     YKLGRKLGSGSFGELFLGVNVQTGEEVAVKLEPARARHPQLHYESKLYMLLQGGTGIPHL 60
CKL13    FKLGRKLGSGSFGEIFLGVNVQTGEEVAVKLEPLRARHPQLHYESKLYMLLQGGTGIPHL 60
CKL10    FKLGRKIGSGSFGELYIGINVQTGEEVALKLEPVKTKHPQLHYESKVYMLLQGGTGVPHI 60
CKL11    FKLGRKLGSGSFGELYLGINIQTGEEVAVKLEPVKTRHPQLQYESKIYMFLQGGTGVPHL 60
CKL7     FKLGKKIGSGSFGELYLGVNVQTGEEVAVKLENVKTKHPQLHYESKLYMLLQGGSGIPNI 60
CKL9     FKLGRKIGSGSFGELYLGINVQTGEEVAVKLESVKTKHPQLHYESKLYMLLQGGTGVPNL 60
CKL6     FKLGRKIGGGSFGELFLAVSLQTGEEAAVKLEPAKTKHPQLHYESKIYMLLQGGSGIPSL 60
CKL3     YKLGRKIGGGSFGEIFLATHVDTFEIVAVKIENSKTKHPQLLYEAKLYRILEGGSGIPRI 60
CKL4     YKLGRKIGGGSFGEIFLATHIDTFEIVAVKIENSKTKHPQLLYEAKLYRTLEGGSGIPRI 60
         ::**:*:*.****::::.  : : *  *:*:*  :: ****  *:::*  ::** *:* :

CKL1     KWFGVEGDYNVLVMDLLGPSLEDLFNFCSRKLSLKSVLMLADQMINRVEFFHSKSFLHRD 120
CKL12    KWFGVEGDYNTLVMDLLGPSLEDLFNFCSRKLSLKSVLMLADQMINRVEYFHSKSFLHRD 120
CKL2     KWYGVEGDYNVLVIDLLGPSLEDLFNFCSRKLSLKTVLMLADQMINRIEFVHQKSFLHRD 120
CKL5     KWYGVEGDYNVLVMDLLGPSLEDLFSYCKRQFSLKTVLMLADQMINRLEFIHSKSYLHRD 120
CK1d     RWCGAEGDYNVMVMELLGPSLEDLFNFCSRKFSLKTVLLLADQMISRIEYIHSKNFIHRD 120
CKL8     KWYGVEGEYNCMVIDLLGPSMEDLFNYCSRRFNLKTVLMLADQMINRVEYMHVRGFLHRD 120
CKL13    KWFGVEGEFNCMVIDLLGPSMEEFFNYCSRSFSLKTVLMLADQMINRVEYMHVKGFLHRD 120
CKL10    KWFGVEGNYNCMAIDLLGPSLEDLFNYCTRSFSLKTVLMLADQLINRVEYMHSRGFLHRD 120
CKL11    KWFGVEGEYSCMVIDLLGPSLEDLFNYCKRIFSLKSVLMLADQLICRVEYMHSRGFLHRD 120
CKL7     KWFGVEGDYSVMVIDLLGPSLEDLFNYCNRKLTLKTVLMLADQLLNRVEFMHTRGFLHRD 120
CKL9     KWYGVEGDYNVMVIDLLGPSLEDLFNYCNRKLSLKTVLMLADQLINRVEFMHTRGFLHRD 120
CKL6     KWFGVQGDYNAMVIDLLGPSLEDLFNYCNRRLTLKAVLMLADQLISRVEYMHSRGFLHRD 120
CKL3     KWFGVDGTENALVMDLLGPSLEDLFVYCGRKFSPKTVLMLADQMLTRIEFVHSKGYLHRD 120
CKL4     RWFGVDGTENALVMDLLGPSLEDLFVYCGRKFSPKTVLMLADQMLTRIEYVHSKGYLHRD 120
         :* *.:*  . :.::*****:*::* :* * :. *:**:****:: *:*:.* :.::***

CKL1     LKPDNFLMGLGRRANQVYIIDFGLAKKYRDSTTHQHIPYRENKNLTGTARYASMNTHLGI 180
CKL12    LKPDNFLMGLGRRANQVHIIDFGLAKKYRDNTTHQHIPYRENKNLTGTARYASMNTHLGI 180
CKL2     IKPDNFLMGLGRRANQVYVIDFGLAKKYRD-SNHQHIPYRENKNLTGTARYASMNTHLGI 179
CKL5     IKPDNFLMGLGRRANQVYIIDYGLAKKYRDSSTHRHIPYRENKSLIGTPRYASLNTHLGI 180
CK1d     VKPDNFLMGLGKKGNLVYIIDFGLAKKYRDARTHQHIPYRENKNLTGTARYASINTHLGI 180
CKL8     IKPDNFLMGLGRKANQVYIIDYGLAKKYRDLQTHRHIPYRENKNLTGTARYASVNTHLGI 180
CKL13    IKPDNFLMGLGRKANQVYIIDYGLAKKYRDLQTHKHIPYRENKNLTGTARYASVNTHLGI 180
CKL10    IKPDNFLMGLGRKANQVYIIDYGLAKKYRDLQTHKHIPYRENKNLTGTARYASVNTHLGI 180
CKL11    IKPDNFLMGLGRRANQVYIIDYGLAKKYKDLQTQKHIPYRENKNLTGTARYASVNTHLGI 180
CKL7     IKPDNFLMGLGRKANQVYIIDFGLGKKYRDLQTHKHIPYRENKNLTGTARYASVNTHLGV 180
CKL9     IKPDNFLMGLGRKANQVYIIDFGLGKKYRDLQTHRHIPYRENKNLTGTARYASVNTHLGV 180
CKL6     IKPDNFLMGLGRKANQVYIIDFGLAKKYRDLQTHRHIPYRENKNLTGTARYASVNTHLGV 180
CKL3     IKPDNFLMGLGRKANQVYLIDFGLAKRYRDANTNRHIPYRENKNLTGTARYASCNTHLGI 180
CKL4     IKPDNFLMGLGRKANQVYLIDFGLAKRYRDANTNRHIPYRENKNLTGTARYASCNTHLGI 180
         :**********::.* *::**:**.*:*:*  .::********.* **.**** *****:

CKL1     EQSRRDDLESLGYILMYFLKGSLPWQGLKAGTKKQKYERISEKKVSTSIEALCRGYPSEF 240
CKL12    EQSRRDDLESLGYILMYFLKGSLPWQGLKAGTKKQKYERISEKKVSTSIESLCRGYPSEF 240
CKL2     EQSRRDDLESLGFVLMYFLKGSLPWQGLKAGNKKQKYEKISEKKVSTSIEALCRGYPSEF 239
CKL5     EQSRRDDIESLGYILMYFLKGSLPWQGLKAGNKKQKYDKISEKKVSTSIETLCRGHPTEF 240
CK1d     EQSRRDDLESLGYVLMYFNLGSLPWQGLKAATKRQKYERISEKKMSTPIEVLCKGYPSEF 240
CKL8     EQSRRDDLESLGYVLMYFLRGSLPWQGLRAGTKKQKYDKISEKKRLTPVEVLCKSFPPEF 240
CKL13    EQSRRDDLESLGYLLMYFLRGSLPWQGLRAGTKKQKYDKISEKKRLTPVEVLCKNFPPEF 240
CKL10    EQSRRDDLESLGYVLMYFIRGSLPWQGLKAGTKKQKYEKISEKKMLTPVEVLCKSYPSEF 240
CKL11    EQSRRDDLESLGYVLMYFLRGSLPWQGLKAGTKKQKYDKISEKKMLTSVETLCKSYPSEF 240
CKL7     EQSRRDDLESLGYVLMYFLKGSLPWQGLKAGTKKQKYDRISEKKVSTPIEVLCKNQPSEF 240
CKL9     EQSRRDDLEALGYVLMYFLKGSLPWQGLKAGTKKQKYDRISEKKVATPIEVLCKNQPSEF 240
CKL6     EQSRRDDLESLGYVLMYFLRGSLPWQGLKAGTKKQKYDRISEKKVSTPIEVLCKSYPPEF 240
CKL3     EQSRRDDLESLGYVLLYFLRGSLPWQGLKAVDKKQKYDKICEKKISTPIEVLCKNHPVEF 240
CKL4     EQGRRDDLESLGYVLLYFLRGSLPWQGLKAVDKKQKYDKICEKKISTPIEVLCKSHPVEF 240
         **.****:*:**::*:**  ********:*  *:***::*.***  *.:* **:. * **

CKL1     ASYFHYCRSLRFDDKPDYAYLKRIFRDLFI 270
CKL12    ASYFHYCRSLRFDDKPDYGYLKRIFRDLFI 270
CKL2     ASYFHYCRSLRFDDKPDYAYLKRLFRDLFI 269
CKL5     ASYFHYCRSLRFDDKPDYAYLKRLFRNLFI 270
CK1d     ATYLNFCRSLRFDDKPDYSYLRQLFRNLF- 269
CKL8     TSYFLYVRSLRFEDKPDYPYLKRLFRDLFI 270
CKL13    TSYFLYVRSLRFEDKPDYSYLKRLFRDLFI 270
CKL10    TSYFHYCRSLRFEDKPDYSYLKRLFRDLFI 270
CKL11    TSYFHYCRSLRFEDKPDYSYLRRLFRDLFI 270
CKL7     VSYFHYCRSLRFDDKPDYSYLKRLFRDLFI 270
CKL9     VSYFRYCRSLRFDDKPDYSYLKRLFRDLFI 270
CKL6     VSYFQYCRSLRFEDKPDYSYLKRLFRDLFI 270
CKL3     ASYFHYCHTLTFDQRPDYGFLKRLFRDLF- 269
CKL4     ASYFHYCHTLTFDQRPDYGFLKRLFRDLF- 269
         .:*: : ::* *:::*** :*:::**:**
```

Supplemental Figure 1. Alignment of kinase domains of Arabidopsis CKL and human CK1delta (CK1d). Leu 85 and Ile 148 that are crucial for B-AZ binding (Figure 4) are marked in orange.
